# Gather your neurons and model together: Community times ahead

**DOI:** 10.1371/journal.pbio.3002839

**Published:** 2024-11-06

**Authors:** Maria Diamantaki, Athanasia Papoutsi

**Affiliations:** 1 Institute of Molecular Biology and Biotechnology, Foundation for Research and Technology-Hellas, Heraklion, Greece; 2 School of Medicine, University of Crete, Heraklion, Greece

## Abstract

Bottom-up, data-driven, large-scale models can provide a mechanistic understanding of neuronal functions. This Primer explores a new study in PLOS Biology that builds a biologically realistic model of the rodent CA1 region that aims to become an accessible tool for the whole hippocampus community.

## Teaser

Bottom-up, data-driven, large-scale models provide a mechanistic understanding of neuronal functions. A new study in *PLOS Biology* builds a biologically realistic model of the rodent CA1 region that aims to become an accessible tool for the whole hippocampal community.

For every neuroscientist out there in the wild, the word “hippocampus” is tightly associated with learning, memory, and spatial navigation. The hippocampus is one of the most studied mammalian brain regions across every possible scale, from molecules and cells to systems, and from anatomical features to functional outputs. It receives wide-spread cortical inputs, transforms them into meaningful representations, and sends the relevant information back to the cortex to guide behavior. Of major importance is the CA1 region that not only works as an integration hub, but also as the output node of the hippocampal circuit [1]. Despite the wealth of literature on CA1, there are still gaps in our knowledge of its function and inconsistencies across experimental findings [2,3]. This is not surprising given that this structure, similar to other brain regions, consists of numerous cell types, embedded in elaborate local and long-range circuits.

The brain’s intricate structure is taken into account in data-driven, large-scale biophysical models. By using an unbiased approach, these models can provide a quantitative framework for inferring the mechanisms that underlie neuronal function. In order to simulate the multitude of biologically realistic features, it is necessary to use diverse and multiscale experimental data to constrain the models. Furthermore, to expand the range of questions a model can address, existing or new experimental data need to be continuously integrated. Likewise, development of these models can probe fragmented experimental data that, along with the model predictions, can be used to guide experiments. Together with advances in high-performance computing, publicly available experimental data sets have instigated the development of such circuit models of different regions, e.g., of the somatosensory [4] and the visual cortex [5]. Based on the cumulative availability of data on the hippocampal structure and function, the first full-scale, publicly shared model of the CA1 region was developed by Bezaire and colleagues [6], revealing new insights into the spatiotemporal organization of the CA1 circuit during theta oscillations. Additionally, the Hippocampome project (hippocampome.org), an organized repository of hippocampal knowledge, was recently extended to build related data-driven spiking neural networks [7]. Despite these prominent efforts, the hippocampal research community has yet to agree on a consensus large-scale data-driven CA1 circuit model to be widely used as a tool.

Romani and colleagues [8] aspire to create such a reference circuit model: the authors performed a tour-de-force and developed an atlas-based biophysically detailed model of the rat CA1 region consisting of different excitatory and inhibitory cell types. Connection properties, such as short-term plasticity and inputs from CA3 to CA1 neurons, were constrained by experimental data, while cholinergic inputs were phenotypically modeled. Importantly, the authors implemented the full 3D structure of the CA1 region, namely by incorporating the local regional geometry—the highly curved shape of the hippocampus—while providing cellular and subcellular specificity. This approach, which compared to abstract 3D spaces mimics more closely the hippocampal anatomy, can further our insights into the spatiotemporal dynamics of the CA1 circuit and allows for interpretation of electrophysiological recordings (e.g., hippocampal curvature and electrode placement for local field potential signals).

Notably, the authors did not tune the model parameters to reproduce specific experimental results and thus envisage that it could be used in a wide spectrum of cases. To showcase how the model can be used to test alternative hypotheses, they performed several simulations examining the hippocampal oscillatory activity under in vitro- and in vivo-like conditions, including the propagation and/or generation of theta, gamma, and delta oscillations. Particular focus was placed on the possible intrinsic (within CA1 generation) and extrinsic (inputs from CA3 or medial septum) mechanisms for the generation of the theta rhythm given its prominence in the hippocampal circuit and the controversy in the literature regarding its supporting mechanism(s) [3,6]. The model’s transparency, reproducibility, and clarity are achieved by (a) providing a detailed methodology for the circuit construction, including a description of the model’s assumptions and the results it fails/succeeds to reproduce; (b) the accessibility of the experimental data, code, and model through the hippocampushub.eu portal, linking to related databases, i.e., to the hippocampome.org [7]; and (c) listing the necessary steps for its expansion to incorporate new data.

Given its public availability, the 3D-atlas-based model of Romani and colleagues [8] can be used in hippocampus research either as a whole, or to simulate slices with desired properties. Targeted manipulations of the model’s parameters could allow the exploration of the underlying neural circuit dynamics in health and disease. It is anticipated that the tools developed for the model construction could be employed for modeling the whole hippocampal formation or other regions. Yet, some important hippocampal features are currently missing from the model, thus restricting the types of questions that can be addressed. For example, the investigation of place cell activity requires significant extension of the model with features like bursty firing of pyramidal neurons, the perforant pathway, and long-term plasticity rules [1]. Similarly, given the atlas-based CA1 structure of the model, integration of the known physiological differences along the dorsal-ventral axis of the CA1 region would be an insightful addition [9].

**Fig 1:**
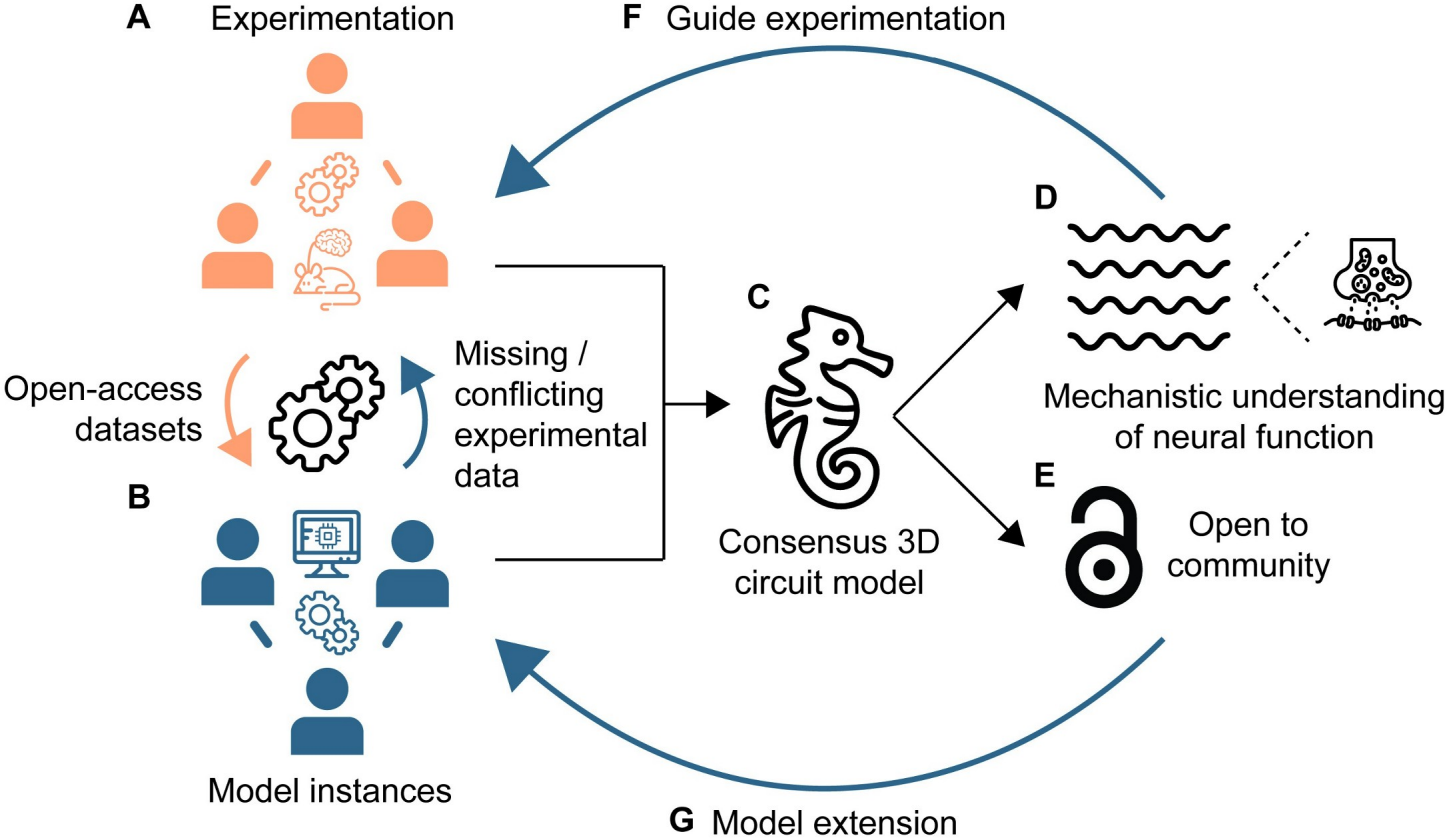
Extended community synergies needed for region-specific, large-scale mechanistic models. Ideal presentation of research community synergistic loop interactions with the aim of shared knowledge acquisition that can drive scientific discoveries. (A) Open-access experimental datasets of different types of information, ranging from molecular to morphological, electrophysiological, and anatomical data as well as data sets linking neural activity to cognitive functions are used for the development of a pool of model instances. (B) During model creation, modelers identify fragmented experimental data and, along with model predictions inform experimentalists. (C) Cumulative knowledge can be combined through the synergy within and beyond the computational community to produce a consensus model, e.g., a large-scale CA1 circuit model that can be used to address multiple hypotheses rigorously. (D) As an example, targeted manipulations of the model parameters provide a mechanistic understanding of the emergence of CA1 theta oscillations. (E) The FAIR principles are a cornerstone of these efforts. (F) The consensus model can inform and design experiments, implementing the 3Rs in animal research. Results from the experiments feedback and refine the models further increasing their predictive power. (G) User-friendly, public accessibility of the model will allow researchers to reuse, question, and extend it, potentially generating different variations of the model to address specific questions. These variations become part of the pool of the model instances to be integrated into an updated consensus model. Waves icon by Yosua Bungaran, synapse icon by dDara, seahorse icon by ProSymbols, licensed under CC BY 4.0 from thenounproject.com. All other icons are adapted with No Copyright from www.svgrepo.com.

Concluding, for the efficient uptake of this or similar models by the hippocampal community a closed-loop synergy between the experimental and computational communities is imperative (Fig 1): First, biologically realistic models developed to address specific hypotheses (model instances) need to transverse levels of abstraction, from single-neuron biophysics to circuit dynamics, and thus incorporate a lot of details. This necessitates the availability of standardized, open-access experimental data sets of different types of information (Fig 1A) and a carefully designed procedure for their integration into models. During the creation of model instances (Fig 1B), modelers identify missing or conflicting experimental data that, along with model predictions, close the first synergistic loop. Second, extensive collaborations within and beyond the computational community can produce a consensus model (Fig 1C). This consensus model is more generic in the questions/hypotheses it can address since it integrates multiple experimental data and computational models, and can thus be widely used by the research community. As such, it is expected to provide a rigorous quantitative framework to infer the mechanisms that underlie neural dynamics (Fig 1D). Moreover, robust model predictions are used to inform and design experiments, implementing the 3Rs (Replacement, Reduction, Refinement) in animal research, closing the second synergistic loop (Fig 1F). Finally, applying the FAIR principles (Findability, Accessibility, Interoperability, and Reuse, Fig 1E) to models and modeling workflows, in addition to the data used to constrain them, allows researchers to reuse, question, and extend published models [10] (Fig 1G), closing the third synergistic loop.

A consensus model makes sense only if it finds its way back to the community; in any other case, it is a model instance. In that regard, we see the work of Romani and colleagues, as complementary to similar efforts in the CA1 region [6,7] and we believe that strong ties with the current community should be established or, when already in place, expanded. A true synergy within and beyond the computational neuroscience community can only exploit and advance scientific discoveries. In short, may all the different models come in unison.
